# Case Report: Pansteatitis with sterile nodular panniculitis (SNP) in a dog

**DOI:** 10.3389/fvets.2026.1681878

**Published:** 2026-02-20

**Authors:** Jihee Han, Yenah Lee, Youngsin Seung, Kunho Song

**Affiliations:** 1 Department of Veterinary Internal Medicine, College of Veterinary Medicine, Chungnam University, Daejeon, Republic of Korea; 2 Time Animal Medical Center, Daejeon, Republic of Korea

**Keywords:** fat, inflammation, panniculitis, pansteatitis, sterile nodular panniculitis

## Abstract

A 3-year-old, castrated male Jindo dog presented with a 5-day history of pyrexia, lethargy, anorexia, and abdominal pain. Physical examination revealed multiple subcutaneous nodules, and abdominal ultrasonography showed numerous intra-abdominal nodules. Computed tomography (CT) identified widespread subcutaneous fatty nodules with fat stranding and multiple poorly defined nodular lesions within the abdominal fat. Histopathological examination confirmed panniculitis and steatitis, with no infectious agents identified. Based on these findings, a final diagnosis of sterile nodular panniculitis (SNP) and pansteatitis was made, and the dog was treated with glucocorticoids. Follow-up CT performed 4 months after the initiation of therapy showed marked improvement in the subcutaneous nodules, with only a few residual intra-abdominal lesions remaining. As the dog remained clinically asymptomatic, glucocorticoid therapy was discontinued. At the time of writing, no clinical recurrence had been observed. This is the first reported case in which sterile nodular panniculitis and pansteatitis were diagnosed and treated using minimally invasive approaches, including whole-body computed tomography and laparoscopic biopsy.

## 1 Introduction

Panniculitis is an inflammation of subcutaneous fat (1). It can result from various underlying causes, including pancreatitis, pancreatic tumors, foreign bodies, immune-mediated reactions, infections, vitamin E deficiency, serum alpha-1-antitrypsin deficiency, injection reactions, burns or trauma (2).

Sterile nodular panniculitis (SNP) is characterized by sterile subcutaneous inflammatory nodules that may ulcerate and discharge a purulent or oily exudate. In dogs with SNP, 20 ~ 86% present with multiple lesions and systemic clinical signs, such as pyrexia, poor appetite, depression and lethargy, are more frequently observed in these dogs (1, 3, 4).

Pansteatitis is a marked inflammation of adipose tissue accompanied by deposition of ceroid pigment in fat cells. This condition may occur in cats when the dietary content of vitamin E is insufficient or when a diet contains high levels of polyunsaturated fatty acids, which can deplete vitamin E (5). Pansteatitis may also arise from similar underlying causes as panniculitis (6). The predominant clinical manifestations of pansteatitis include fever, lethargy, anorexia, and pain elicited on palpation of subcutaneous nodular lesions (7).

Although pansteatitis has been documented in cats, reports in dogs are rare. This is the first report of a case in which SNP and pansteatitis were diagnosed and treated based on whole-body CT and histopathological examination, including laparoscopic biopsy.

## 2 Case presentation

A 3-year-old, 24.5 kg, castrated male Jindo dog presented with a 5-day history of pyrexia, lethargy, anorexia, and abdominal pain. The dog had been regularly vaccinated and dewormed. No gastrointestinal signs, such as vomiting or diarrhea, were reported.

On physical examination, the dog had a body condition score of 4/9. Mucous membranes were moist and pink, and skin turgor was normal, indicating no evidence of dehydration. The heart rate was 180 beats per minute, the respiratory rate was elevated with panting, and rectal temperature was 40.5 °C, indicating marked pyrexia. Multiple subcutaneous nodules were identified; these were firm on palpation and non-pruritic. There was no history of subcutaneous injection or vaccination at the affected site. Mild symmetrical enlargement of the prescapular, axillary, and popliteal lymph nodes was observed.

Hematological analysis (ProCyte Dx^®^, IDEXX Laboratories, Westbrook, ME, USA) revealed a total white blood cell count (WBC) within the reference range at 15.73 × 10^9^/L (RI: 5.05–16.76 × 10^9^/L); however, eosinophilia was noted with an eosinophil count of 3.73 × 10^9^/L (RI: 0.06–1.23 × 10^9^/L).

Serum biochemistry (BS-240 Vet, Mindray Animal Medical Technology Co., Ltd.) showed mild elevations in alkaline phosphatase (ALP), at 266.13 U/L (RI: 15–127 U/L), and aspartate aminotransferase (AST), at 92.74 U/L (RI: 15–43 U/L). C-reactive protein (CRP) was markedly increased at 11.83 mg/dL (RI: 1–2 mg/dL), while canine pancreatic lipase (cPL) was within normal limits at 50 ng/mL (RI: 200–400 ng/mL). Ionized calcium concentration was within the reference range at 1.4 mmol/L (RI: 1.14–1.40 mmol/L), urinalysis revealed no significant abnormalities. The reference intervals for each parameter were derived from the manufacturer’s canine reference data provided by the analyzers.

Radiographs identified several uroliths within the urinary bladder, with no other significant abnormalities noted.

**Figure 1:**
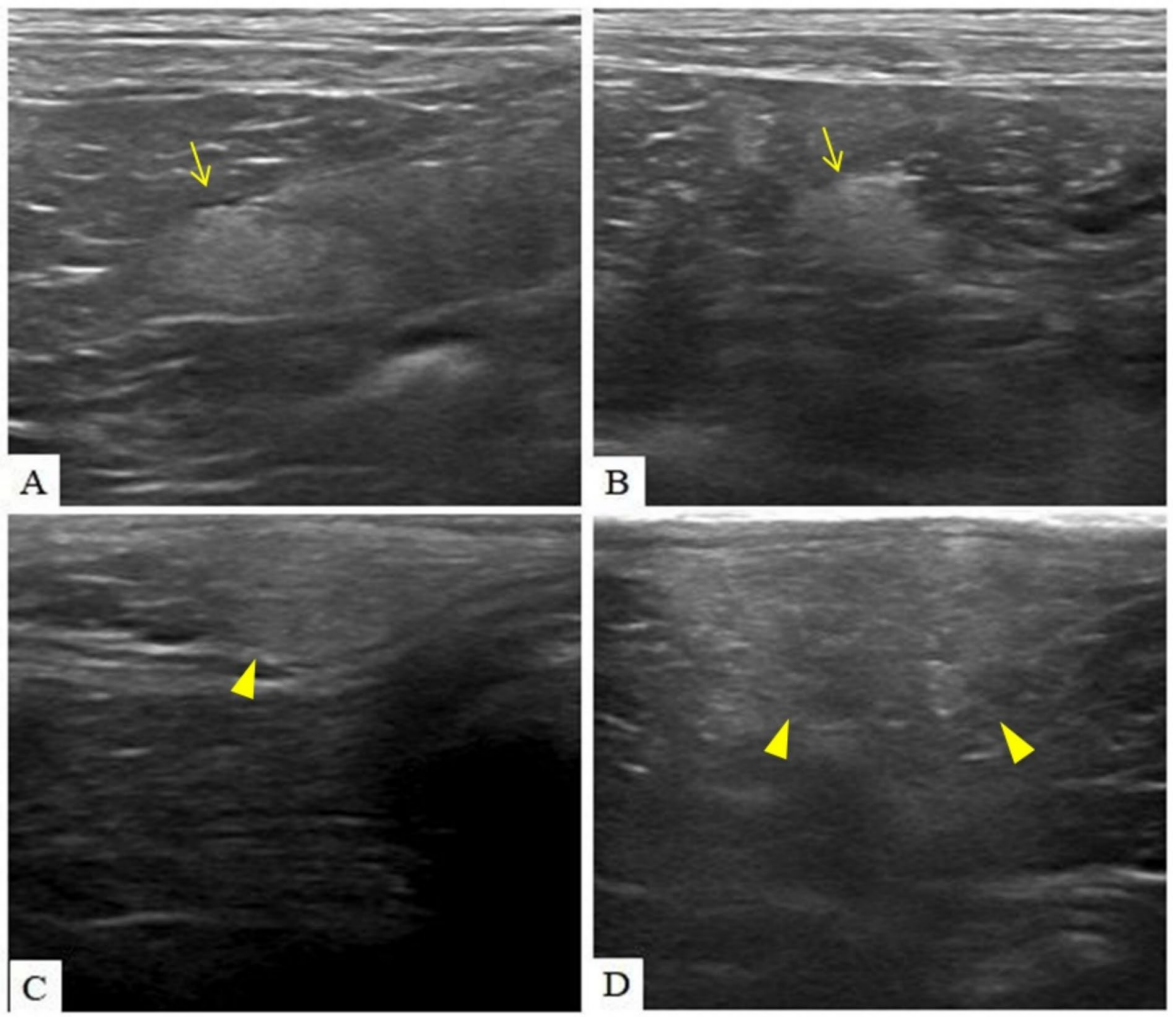
Multiple diffusely distributed, poorly marginated hyperechoic nodules (arrow) were observed throughout the peritoneum, accompanied by increased overall peritoneal echogenicity **(A,B)**. Ill-defined hyperechoic nodules (arrow head) extended across the dermis, subcutaneous tissue, and underlying muscle **(C,D)**. The nodules identified in the peritoneal cavity and subcutaneous tissue exhibited comparable echogenicity and morphological feature.

Abdominal ultrasonography revealed diffusely distributed, poorly marginated hyperechoic nodules throughout the peritoneum, accompanied by increased peritoneal echogenicity and a small volume of peritoneal effusion. Ultrasonographic examination of the skin revealed multiple ill-defined hyperechoic nodules extending across the dermis, subcutis, and underlying muscle (Figure 1).

Supportive treatment, including antibiotic therapy, was initiated. However, by day 5 of hospitalization, the dog continued to have a high fever, and, CRP levels remained elevated at 11.07 mg/dL. Additionally, ionized calcium concentration had increased to 1.74 mmol/L.

Polymerase chain reaction (PCR) testing for vector-borne and systemic infectious diseases returned negative results for all of the following organisms: *Anaplasma* spp., *Babesia canis*, *Babesia gibsoni*, *Bartonella* spp., *Blastomyces dermatitidis*, *Brucella canis*, *Coccidioides* spp., *Ehrlichia* spp., *Hepatozoon* spp., *Histoplasma capsulatum*, *Leptospira* spp., *Neospora* spp., *Rickettsia rickettsii*, *Toxoplasma gondii*, *Trypanosoma cruzi*, and Severe Fever with Thrombocytopenia Syndrome (SFTS) virus.

Ultrasound-guided fine-needle aspiration (FNA) of the subcutaneous nodules was performed using a 22-gauge needle under real-time ultrasonographic visualization to avoid vascular structures and obtain representative cellular material (8). Cytological evaluation revealed numerous neutrophils and a small number of lymphocytes; however, the cellularity was low. The dog underwent computed tomography (CT; SOMATOM Scope, Siemens Healthcare, Erlangen, Germany; 130 kVp, 100 mAs, 2 mm slice thickness, 0.6 s tube rotation time, pitch 0.6) and surgical biopsy. Samples of the peritoneal nodules were obtained via laparoscopy, while subcutaneous nodules were collected through subcutaneous excision.

**Figure 2:**
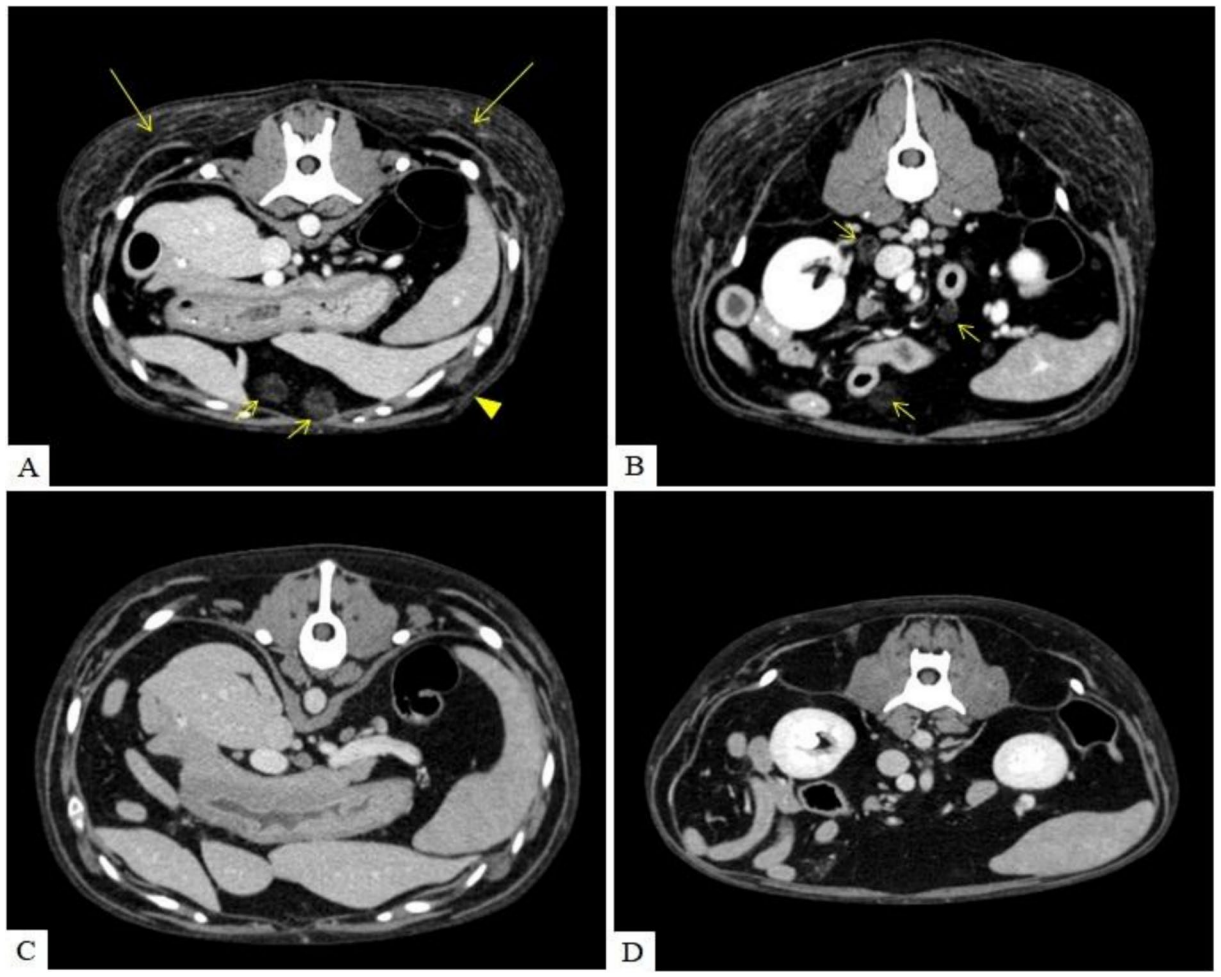
Transverse CT images of the abdomen before treatment **(A,B)** and 4 months after treatment **(C,D)**. Before treatment, fat stranding was observed in the subcutaneous tissue (long arrow). Multiple poorly marginated nodules exhibiting contrast enhancement were identified within the subcutaneous fat (arrow head). Similar nodules, up to 2.3 cm in diameter and with fat attenuation (approximately −60 HU), were also noted in the intra-abdominal fat, with some showing mild post-contrast enhancement (short arrow). CT performed 4 months after treatment **(C,D)** showed that most of the subcutaneous fat stranding had resolved. The majority of the subcutaneous and intra-abdominal nodules had also resolved, and the remaining nodules were markedly reduced in size.

The CT imaging revealed scattered fat stranding throughout the thoracic and abdominal subcutaneous fat, and multiple ill-defined, contrast-enhancing fat nodules were identified in the subcutaneous adipose tissue. While no abnormalities were noted in the pulmonary parenchyma, the cranial mediastinal lymph nodes appeared mildly enlarged with a rounded morphology. Similar to the subcutaneous lesions, multiple fat-density nodules (−60 Hounsfield units), measuring up to approximately 2.3 cm and exhibiting mild contrast enhancement, were also observed within the intra-abdominal fat. Some of the nodules had well-defined margins, whereas others were poorly demarcated. Similar nodules were also present in the retroperitoneal region. The pancreatic parenchyma appeared homogeneous without notable abnormalities (Figure 2).

**Figure 3:**
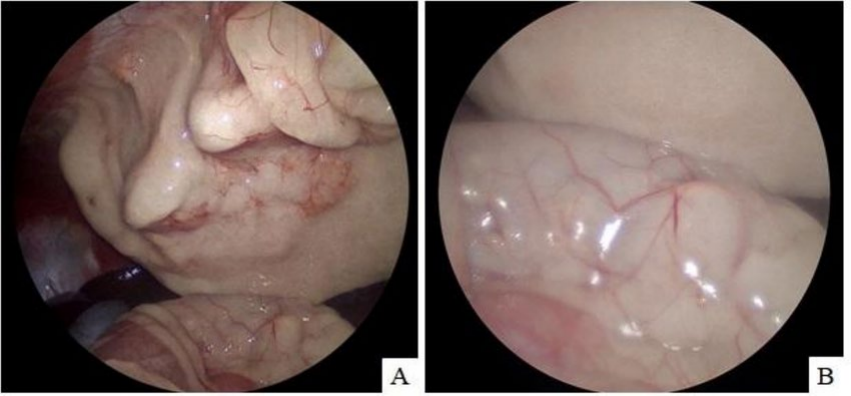
Multiple round masses distributed along the abdominal wall **(A)** and within the falciform fat **(B)**. Some of the masses were excised via laparoscopy for histopathological examination.

Exploratory laparoscopy was performed using a three-port technique. Multiple round masses were observed along the abdominal wall and within the falciform ligament at the midline. Additional masses were identified within the mesenteric fat. Excisional biopsies were then obtained from two separate sites for histopathological evaluation (Figure 3).

While awaiting histopathology results, initial treatment consisted of amoxicillin/clavulanic acid (Amocla^®^, Gunil Pharmaceutical Co., Ltd.) at 22 mg/kg, PO, BID; prednisolone (Solondo^®^, Yuhan Corporation) at 0.3 mg/kg, PO, SID; famotidine (Famotidine^®^, Nelson Pharmaceutical Co., Ltd.) at 0.5 mg/kg, PO, BID; silymarin (Silymarin^®^, ELT Science) at 5 mg/kg, PO, BID; and ursodeoxycholic acid (URSA^®^, Daewoong Pharmaceutical Co., Ltd.) at 5 mg/kg, PO, BID. The dog’s fever resolved on the second day after glucocorticoid administration.

**Figure 4:**
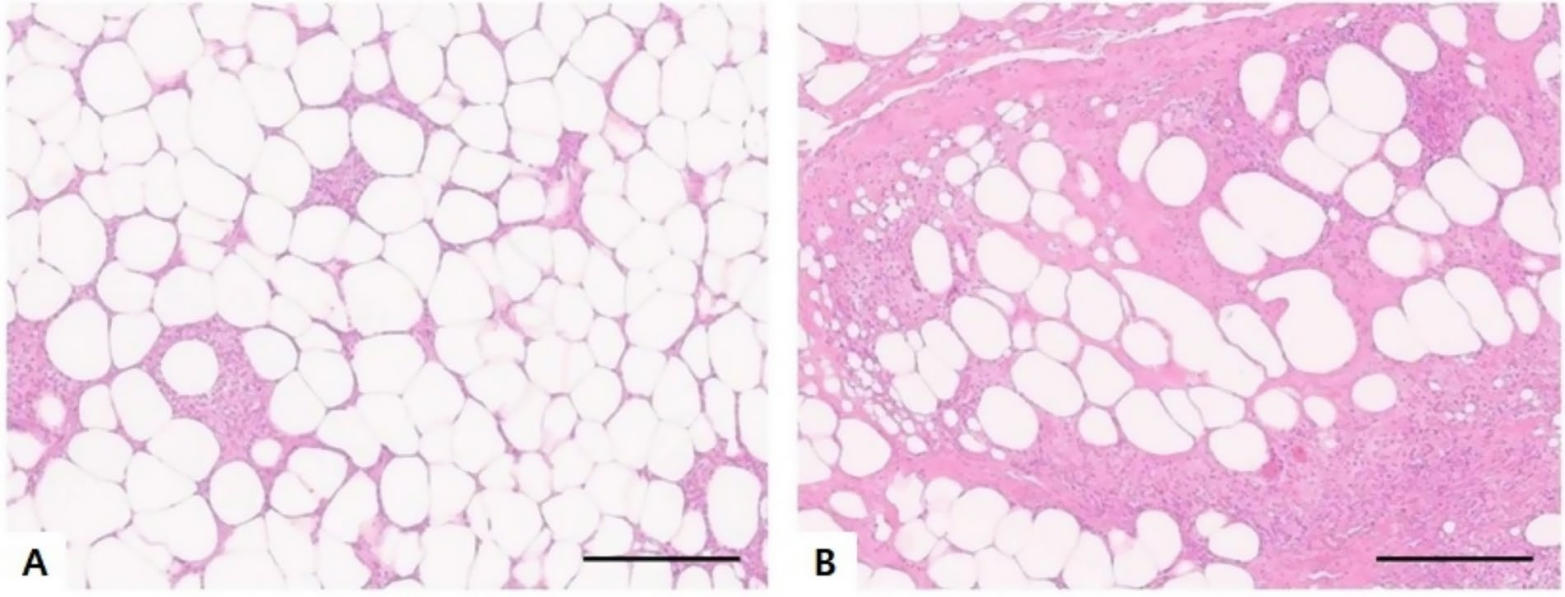
Histopathological examination of the intra-abdominal mass **(A)** and subcutaneous mass **(B)**. Both tissues were of adipose origin and exhibited granulomatous inflammation composed of macrophages, neutrophils, and a few lymphocytes, accompanied by fibrosis (HE; magnification, ×200 in **A,B**; scale bars, 100 μm).

Histopathological examination of the subcutaneous and abdominal nodules revealed multifocal to coalescing severe inflammation with septal fibrosis. The predominant inflammatory cells included macrophages, lymphocytes, several multinucleated giant cells, and neutrophils. No evidence of neoplasia was observed in either lesion (Figure 4), and, bacterial and fungal growth was not observed following culture of the abdominal nodules.

The dog was diagnosed with pansteatitis and SNP. Upon re-evaluation 9 days later, rectal temperature was within the normal range (38.8 °C), and the patient showed improved vitality and appetite. Accordingly, antibiotic therapy was discontinued. At a follow-up visit 18 days post-diagnosis, the dog remained clinically well. CRP was measured at 0.48 mg/dL and ionized calcium at 1.4 mmol/L, within the normal range, and no fever was detected on physical examination.

Prednisolone was tapered to 0.25 mg/kg SID and continued for an additional 2 weeks. The subcutaneous nodules on the dorsum completely resolved, and those on the cranial thoracic and shoulder regions decreased in size. However, due to insufficient regression of the remaining nodules, the prednisolone dose was increased to 1 mg/kg BID, followed by biweekly tapering. Complete resolution of the subcutaneous nodules was observed at a prednisolone dose of 0.75 mg/kg BID, and no recurrence of nodules was noted up to the point when the dose was reduced to 0.3 mg/kg BID.

Four months after the initiation of glucocorticoid therapy, a follow-up CT scan was performed. Most of the subcutaneous fat stranding had resolved, with only mild residual changes observed in the gluteal and femoral regions. The subcutaneous nodules had significantly improved, though a few remained in reduced size, and mild enlargement of the cranial mediastinal lymph node was noted. Most abdominal nodules had resolved, with the remaining lesions showing marked size reduction (Figure 4). Despite incomplete resolution of all lesions, the owner reported that the patient showed marked improvement in appetite, activity level, and overall comfort shortly after starting treatment.

Consequently, prednisolone was discontinued, and no evidence of recurrence was observed at the time of writing, with the patient remaining free of clinical relapse for over 5 months after the initiation of therapy.

## 3 Discussion

Sterile nodulr panniculitis and pansteatitis exhibit similar histopathological features, including infiltration of numerous macrophages and neutrophils within fibrinous tissue. Their underlying pathogenesis is also considered to be similar (6, 9).

Several reports have described the concurrence of pancreatic disease and panniculitis. For instance, concurrent pancreatitis and panniculitis have been reported in Cocker Spaniels (10). In two dogs with pancreatic neoplasia, panniculitis was accompanied by polyarthritis, and in a cat with pancreatic adenocarcinoma, both cutaneous and visceral necrotizing panniculitis were documented (11, 12).

Pancreatic diseases result in elevated levels of pancreatic enzymes in the plasma, which can induce saponification of lipid-rich tissues such as subcutaneous fat and bone marrow. These enzymes can enter the systemic circulation via the thoracic duct, portal venous system, or lymphatic channels (13). Additionally, in cases of alpha-1 antitrypsin deficiency or reduced levels of alpha-2 macroglobulin, the decreased inhibition of trypsin activity may lead to elevated concentrations of free trypsin in the bloodstream, which can contribute to the development of panniculitis lesions (10, 13).

In the present case, aside from a mild elevation in serum amylase, both lipase and cPL levels were within the normal range. Moreover, abdominal ultrasonography and CT revealed a homogeneous pancreatic parenchyma without any notable abnormalities. These findings suggest that the coexistence of pancreatitis and pancreatic neoplasia was unlikely.

In one study, approximately 40% of dogs diagnosed with sterile panniculitis were also affected by atopic dermatitis (9). However, no immune-mediated disease was identified in the present case. In human medicine, there have been reports of autoimmune cholangitis and pancreatitis associated with panniculitis in the context of IgG4-related disease, managed with analgesics, antibiotics, and prolonged corticosteroid therapy (14). Additionally, lupus erythematosus panniculitis is a rare variant of cutaneous lupus erythematosus, occurring in approximately 2–5% of patients with systemic lupus erythematosus (15). However, to date, no studies have documented the concurrent occurrence of pansteatitis and immune-mediated disease in veterinary medicine, except in association with atopic dermatitis.

In another study, adipose tissue in the panniculus or deep subcutis was inflamed, primarily by neutrophils and macrophages, and surrounded by fibrous connective tissue in 70% cases (6, 8). In the present case, histopathological examination revealed granulomatous lesions consisting predominantly of macrophages and neutrophils, with a small number of lymphocytes. These findings are consistent with previously reported the microscopic features of sterile nodular panniculitis and pansteatitis. Furthermore, both the subcutaneous and intra-abdominal nodules exhibited similar histopathological characteristics.

Accordingly, treatment with glucocorticoids was initiated as in previous reports, and the patient showed clinical improvement in response to therapy. In previous studies, all cases responded to glucocorticoid therapy, although some dogs experienced relapse following steroid tapering (6). In the present case, clinical improvement was achieved with glucocorticoids alone, that is, without the use of additional antibiotics, antifungals, or antiparasitic agents, suggesting a strong immune-mediated component in the pathogenesis of this disease. Given this, further research into the immune-mediated mechanisms involved is warranted. In the event of relapse, combination therapy with other immunosuppressants, such as azathioprine or cyclosporine, may be required (1).

At initial presentation, the patient’s serum ionized calcium concentration was at the upper limit of the normal range; however, on day 5 of hospitalization, the ionized calcium level increased to 1.74 mmol/L. The subcutaneous nodules remained palpable, and the patient exhibited persistent fever with a body temperature of 40 °C.

According to a previous study, histopathological examination of six dogs diagnosed with granulomatous or pyogranulomatous steatitis revealed elevated ionized or total calcium concentrations. Although a definitive causal relationship between steatitis and hypercalcemia has not been established, in cases without identifiable causes of hypercalcemia, full-thickness skin biopsies performed before and after corticosteroid treatment showed resolution of granulomatous lesions accompanied by normalization of blood calcium levels (16). This phenomenon is thought to arise from increased activity of macrophage-derived 1-*α*-hydroxylase during pyogranulomatous inflammation, which converts 25-hydroxyvitamin D to 1,25-dihydroxyvitamin D, enhancing calcium absorption in the intestines and kidneys (17–19). Although parathyroid hormone (PTH) and parathyroid hormone-related protein (PTHrP) levels were not measured in the present case, the concurrent improvement of inflammation and normalization of ionized calcium levels support the hypothesis that macrophage-mediated overproduction of 1-*α*-hydroxylase contributed to the hypercalcemia observed in this case.

Pansteatitis has been reported in cats as a result of a dietary imbalance between vitamin E and unsaturated fatty acids. Vitamin E acts as a crucial antioxidant, protecting lipids from oxidative damage; therefore, diets deficient in vitamin E and high in unsaturated fatty acids can lead to lipid peroxidation, resulting in inflammation of adipose tissue and adipocyte necrosis (20). In dogs, increased dietary intake of polyunsaturated fatty acids has also been shown to correlate with decreased serum tocopherol concentrations (21). In the present case, the dog had been fed a commercially available, nutritionally balanced diet; however, serum tocopherol levels were not measured. Nevertheless, empirical supplementation with vitamin E was initiated.

In conclusion, sterile steatitis involving both subcutaneous and intra-abdominal fat can occur in dogs, presenting with systemic clinical signs such as fever, lethargy, and inappetence.

CT and histopathological examination via biopsy are essential for diagnosis. Histologically, the condition is characterized by infiltration of numerous neutrophils and macrophages with accompanying fibrosis. Infectious causes must be ruled out through bacterial and fungal cultures as well as special staining techniques. Treatment involves systemic administration of glucocorticoids at immunosuppressive doses.

## Data Availability

The raw data supporting the conclusions of this article will be made available by the authors, without undue reservation.
